# Contactless assessment of heart rate in neonates within a clinical environment using imaging photoplethysmography

**DOI:** 10.3389/fped.2024.1383120

**Published:** 2024-04-12

**Authors:** Libor Svoboda, Jan Sperrhake, Maria Nisser, Luca Taphorn, Hans Proquitté

**Affiliations:** ^1^ Department of Pediatric and Adolescent Medicine, University Hospital Jena, Jena, Germany; ^2^ Xsight Optics GmbH, Jena, Germany

**Keywords:** contactless monitoring, non-invasive, imaging photoplethysmography, heart rate, newborns, vital parameters, neonatology, preterm infants

## Abstract

**Introduction:**

In neonatology, the accurate determination of vital parameters plays a pivotal role in monitoring critically ill newborns and premature infants, as well as aiding in disease diagnosis. In response to the limitations associated with contact-based measurement methods, substantial efforts have been directed toward developing contactless measurement techniques, particularly over the past decade.

**Methods:**

Building upon the insights gained from our pilot study, we realized a new investigation to assess the precision of our imaging photoplethysmography-based system within a clinical environment of the neonatal intermediate care unit. We conducted measurements in 20 preterm infants or newborns requiring therapeutic interventions. As a point of reference, we employed a conventional pulse oximeter. To analytically predict measurement artifacts, we analyzed the potential influence of confounding factors, such as motion artifacts, illumination fluctuations (under- and overexposure), and loss of region of interest prior to heart rate evaluation. This reduced the amount of data we evaluated for heart rate to 56.1% of its original volume.

**Results:**

In artifact-free time segments, the mean difference between the pulse oximetry and the imaging photoplethysmography-based system for 1 s sampling intervals resulted in −0.2 bpm (95% CI −0.8 to 0.4, LOA ± 12.2). For the clinical standard of 8 s averaging time, the mean difference resulted in −0.09 bpm (95% CI −0.7 to 0.6, LOA ± 10.1). These results match the medical standards.

**Discussion:**

While further research is needed to increase the range of measurable vital parameters and more diverse patient collectives need to be considered in the future, we could demonstrate very high accuracy for non-contact heart rate measurement in newborn infants in the clinical setting, provided artifacts are excluded. In particular, performing *a priori* signal assessment helps make clinical measurements safer by identifying unreliable readings.

## Introduction

Determining vital parameters is a crucial aspect of neonatology and broader medical practice. One fundamental parameter assessed is the heart rate (HR), which yields critical clinical information. HR assessment commences shortly after birth, providing initial insights into postnatal adaptation success. Resuscitation may be warranted if HR falls below specific thresholds (1, 2).

Compromised newborns, especially preterm infants, necessitate continuous vital parameter monitoring. Electrocardiography (ECG) serves as the gold standard for HR determination, particularly shortly after birth, offering the most rapid and accurate measurement (3–5). Pulse oximetry-based photoplethysmography (PPG), employed for arterial oxygen saturation (SpO_2_) and HR determination, may exhibit slight delays compared to ECG, especially during neonatal resuscitation (6). Typically, PPG signals within neonatal intensive care units (NICUs) use 8 s default averaging time to optimize data accuracy and relevance (7).

Both methods usually involve contact-based sensors, presenting general and age-related drawbacks. Insufficient perfusion, as seen in vasoconstriction or hypotension, may render pulse oximetry-based sensors ineffective (8, 9). Additionally, pain, skin irritation, pressure ulcers, and infections caused by adhesive electrodes or sensors are notable concerns, particularly for premature infants (10–15). Additionally, Anton et al. 's review (16) suggests that several studies have shown that the time between birth and successful establishment of HR using ECG or PPG frequently exceeds 1–2 min.

In response to these limitations and the diverse applications of vital parameter monitoring, research on contactless monitoring methods has intensified, primarily over the last decade. Many non-contact HR measurement methods rely on imaging photoplethysmography (iPPG) principles (17, 18). The iPPG method derives HR measurements by employing the same fundamental principles as conventional pulse oximetry-based photoplethysmography (PPG), which entails the detection and amplification of subtle cyclic changes in skin color associated with each cardiac cycle. Several studies, including our pilot study (19), have demonstrated the feasibility of contactless HR measurement in neonates using iPPG (11–13, 19–28). However, it is essential to note that the iPPG signal is susceptible to various sources of interference, including motion artifacts, changes in ambient light, skin pigmentation variations, and obstruction of the region of interest (ROI) by other equipment or healthcare professionals (4, 11, 17, 22, 25, 29).

In our pilot study (19), we evaluated the accuracy and feasibility of a multimodal 3D camera system using iPPG to measure HR in healthy term and late preterm infants under standardized conditions. Our approach, incorporating 2D and 3D vision, demonstrated feasibility when compared to a reference (*Masimo Rad-97 Pulse CO-Oximeter®*). Notably, the 2D-based method showed a mean difference of only +3.0 bpm compared to the reference, while the 3D-based method revealed a mean difference of +8.6 bpm. Due to accuracy and data volume considerations, we chose to focus on 2D-based methods and discontinuing 3D imaging.

This study aims to validate the accuracy and feasibility of an adapted camera-based system for assessing HR in preterm or compromised newborn infants within the neonatal intermediate care unit (IMC) clinical environment. Additionally, the study seeks to identify and quantify potential disruptive factors to increase the robustness and suitability of iPPG-based HR measurement for everyday use.

## Materials and methods

### Structure and function of the camera sensor

The iPPG camera system utilized in this study is a simplified version of the multi-modal setup implemented in our pilot study. This system consists of two near-infrared (NIR) Genie Nano M1280-NIR cameras equipped with filters at 750 nm and 950 nm, along with a standard RGB Bayer-pattern Genie Nano C1280-IRC camera for color sensing. All three cameras recorded at a frame rate of 30 Hz. To adjust brightness, we turned on ceiling lights when there was insufficient sunlight, relying on ambient illumination as a visible light source. As ceiling lights emit low intensity at 750 nm and no detectable intensity at 950 nm, the system includes three light-emitting diodes (LEDs). Two of these LEDs operate at 950 nm, which is undetectable by the human eye, while the other operates at 750 nm, which is barely visible.

**Figure 1:**
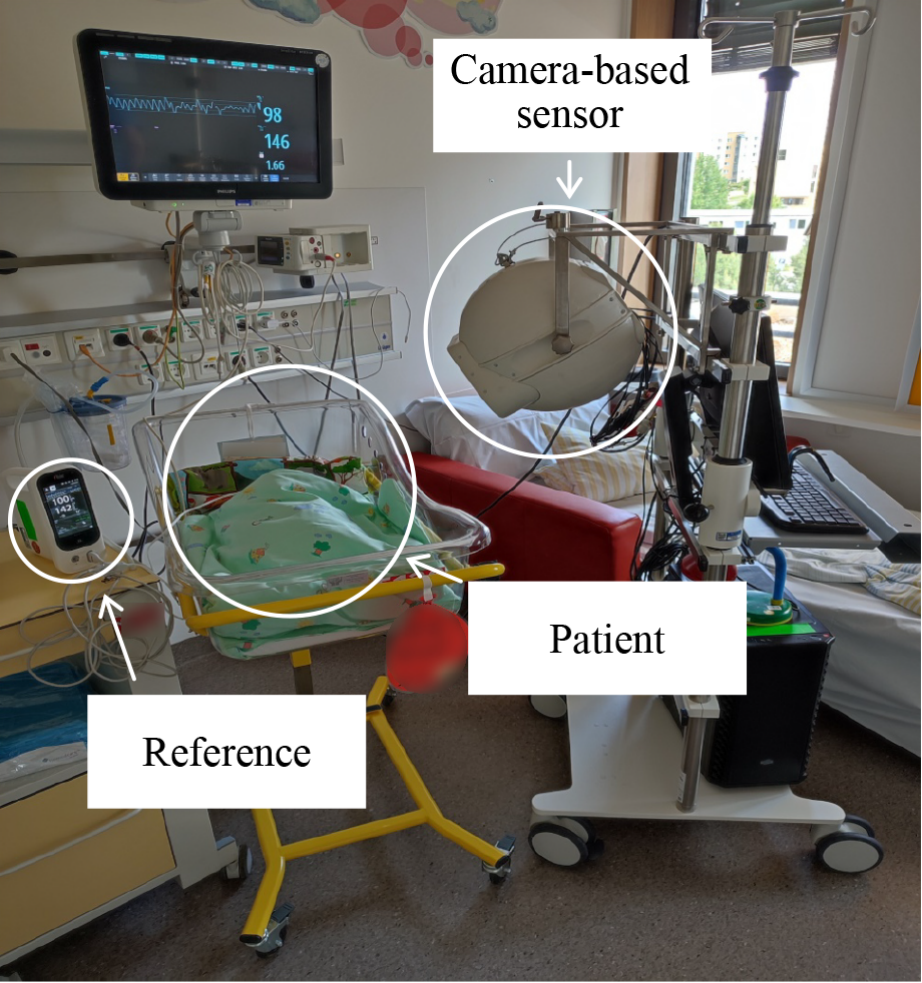
The camera-based sensor deployed within the neonatal intermediate care ward (IMC) positioned close to the patient's bed during the data acquisition process.

The cameras were arranged in a horizontal line, with the RGB camera in the center and the 950 nm and 750 nm cameras on its left and right, respectively. The RGB camera sets a fixed optical axis normal to the front plane of the sensor. The two NIR cameras were tilted towards the optical axis to center their field of view on a measurement target at a distance of 50 cm. The LED lights were located directly beneath the camera array, with the 950 nm LEDs located on the outside and the 750 nm LED positioned in the center. The geometric arrangement in this study was identical to that shown in Figure 1 of our pilot study (19).

Our primary source of image data for iPPG HR evaluation was the RGB camera. The two near-infrared (NIR) cameras provided supplementary image data regardless of ambient light. Additionally, the NIR data gathered in this study serves as a foundation for possible iPPG SpO_2_ assessments, given that the 750 nm and 950 nm wavelengths encompass the point where the extinction coefficients of oxygenated and deoxygenated hemoglobin intersect (30). Although the study did not aim to assess SpO_2_ levels due to the absence of patients with significant oxygen saturation deviations, we collected data for potential follow-up research.

The camera system was securely enclosed in a 3D-printed spherical shell that housed essential electronics for device control and protection. The cameras transmitted the data via Ethernet to an auxiliary computer for data storage. All elements of the system were mounted onto a medical cart (Figure 1) to enable mobility. Compared to our previous wall-mounted setup in the pilot study, our new setup provided us with the freedom to move between rooms and adjust the camera.

### A priori evaluation of the measurement condition

Prior to estimating and analyzing any HR data, we evaluated the reliability of each recording based on the properties of the ROI. By processing the collected data in this order, we avoided bias toward physiological plausibility. The *a priori* evaluation of the ROI involved three characteristics: motion within the ROI, illumination of the ROI, and validity of the ROI (as explained further). All of which are based on color averages of the ROI.

Motion was determined by the temporal standard deviation of the color signals within an interval, weighted by their respective mean, giving a threshold for motion within the ROI. If the motion was too high within a ROI, the corresponding time intervals were denoted as invalid. Next, the illumination of a ROI was classified by minimal and maximal boundary conditions of average color values within a time interval. If values were below or above the boundaries, the interval was labeled invalid. Finally, the validity of the ROI is a measure whether the fixed image box still contains the desired region of interest of the patient. An invalid ROI could be caused by movement or occlusion. It was measured as the angular difference in color space between the average color of the ROI within a time interval and the global color average of the ROI. If the difference exceeded a threshold value, the ROI was identified as inaccurate, and the corresponding time interval was labeled invalid.

**Figure 2:**
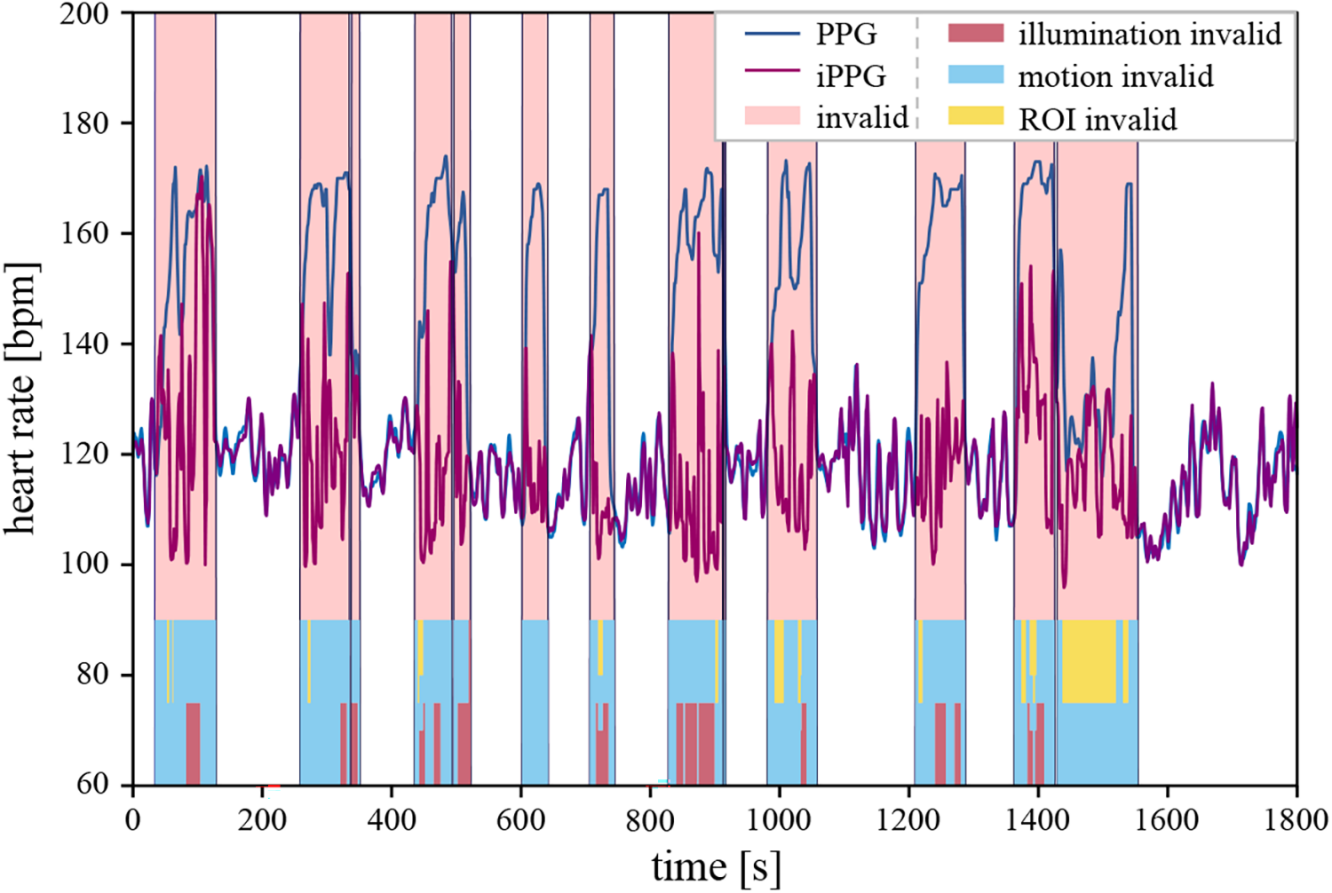
A graphical representation of measurement including heart rate curves from both systems (blue line PPG, purple line iPPG), with light red intervals marking invalid segments, which were excluded from the subsequent statistical analysis. The color bars within the segments at the bottom of the plot indicate the identified disruptive factors. Red, blue, and yellow denote invalid illumination, motion, and ROI conditions, respectively. Multiple colors are shown in the plot if more than one disruptive factor was identified over a time segment.

If any of these properties were invalid, the corresponding time intervals were discarded from the HR evaluation. An example measurement from this study is shown in Figure 2, comparing the reference to the iPPG results while segmenting the measurement into valid and invalid intervals.

### Heart rate estimation from camera images

To extract HR-related signals from the video recordings of patients acquired with our camera system, we used bounding boxes as regions of interest (ROI) that were placed in areas with visible skin pixels. Each recording contained image sequences of a patient's head and upper body. Because head positions and tilt angles of patients' heads varied between measurements, we placed the ROIs manually on the forehead and lateral or posterior head regions. We opted against the tracking of the ROI because it brought no benefits to our approach in this setting. Since most patients had a relatively stable resting position, selecting a fixed bounding box was an adequate choice. Each evaluation involved averaging the pixel values within the ROI for each color channel of each frame and combining them to a 3-dimensional vector in color space, which served as the basis for signal calculation. The time sequence of color vectors was split into small time windows with temporal overlap. The pulse wave signal was then retrieved for each time window using XminaY demixing from the chrominance-based method described in (31).

Initially, we estimated a temporary HR corresponding to the expected value of a normal distribution adapted to the power spectrum of the pulse wave signal. The reliability of the estimate was measured using the width of the distribution. Subsequently, the HR of the current interval was calculated as a weighted average of several successive estimates. By applying this method to an entire recording, we derived a time-dependent HR signal that could be matched to a reference with comparable sampling intervals.

### Setting and participants

Our research was conducted as a single-center observational study at the University Hospital Jena in Germany. We secured ethical approval for our research from the local ethics committee (Ethics Committee Reference No. 2020-1891-MV), including an amendment dated 19th August 2021. Our clinical trial adhered strictly to the principles outlined in the Declaration of Helsinki and complied with Good Clinical Practice (GCP) guidelines.

The study population comprised cardiopulmonary stable late preterm newborns and full-term compromised newborns at the Neonatal Intermediate Care Ward (IMC) that did not require respiratory support during data collection.

We conducted measurements on a cohort of 20 patients utilizing a simplified version of the camera-based sensor system described in our prior publication (19). Before commencing any measures, we diligently obtained written informed consent from the parents to record image data.

Data collection occurred within the time frame of 19th January 2022 to 30th May 2022. Importantly, data acquisition was executed within the confines of the patients' rooms, eliminating the need to displace them from their familiar surroundings. Throughout the measurement process, patients were positioned either in the embrace of a parent or in their beds, as illustrated in (Figure 1).

We set the standard measurement interval to 30 min, whereby in 3 cases, multiple measurements could be carried out. On the other hand, we terminated measurements in many instances earlier, either because of increasing restlessness of the infant or on parental request. This resulted in a total of around 676 min of video material. The videos were recorded at 30 frames per second, generating a total of 162,000 images for each 30-min measurement interval.

As previously described in the manuscript under “Structure and function of the camera sensor”, the camera sensor was attached to a mobile trolley. The mobile design allowed the system to get the best viewing position. Because the ROIs were localized on a child's head, they did not have to be undressed or moved. The nursing and medical care of the children were also guaranteed at any time.

We positioned the sensor at approximately 50 cm from the child's head. This distance ensured that the child's head was equally visible by all three cameras, given by their respective viewing angle as per the construction of the camera sensor.

We obtained the reference values for HR using a pulse oximeter (*Masimo Rad-97 Pulse CO-Oximeter ®, mcu: 1.068, Prozessor: V 1.4.6.2 i-ss; Techboard: 7e94*). The pulse oximeter sensor was attached to either the patient's wrists or feet. To synchronize the time stamps of measurement intervals from our system with those of the reference, we manually adjusted the system clock of the PC recording the videos to match the system time of the pulse oximeter with an approximate alignment down to a second.

Without the use of an additional external light source, ambient illumination varied between individual measurements. Because measurements were performed in a realistic clinical environment and standard patient rooms, they were particularly exposed to prevailing weather conditions from open window blinds. If the exposure was too low, we switched on the overhead lighting. If the exposure was too high, we lowered the window blinds. However, it was challenging to ensure ideal measurement conditions across all measurements. Therefore, some data was affected by variations in ambient illumination, movement of the patients, and random obstructions of the patient's head.

### Statistical methods

In analyzing the study population, we employed descriptive statistics to summarize the data. For normally distributed data, we presented the mean ± standard deviation. We reported the median along with the 25th and 75th percentiles for non-normally distributed data, specifically HR. Gender distribution was summarized using absolute and relative frequencies.

We utilized a linear mixed model to assess the agreement between the measurements obtained from the Masimo sensor and the camera-based sensor. This model incorporated a random intercept per patient to account for multiple measurements per patient and the associated correlation among these observations. We reported the estimated mean difference between the two systems (Masimo sensor minus camera-based sensor) along with a 95% confidence interval. This mean difference serves as an estimate of bias, indicating the degree of disagreement between the two methods. The 95% confidence interval provides a range of values to quantify the uncertainty associated with this estimate, encompassing possible values for the bias.

**Figure 3:**
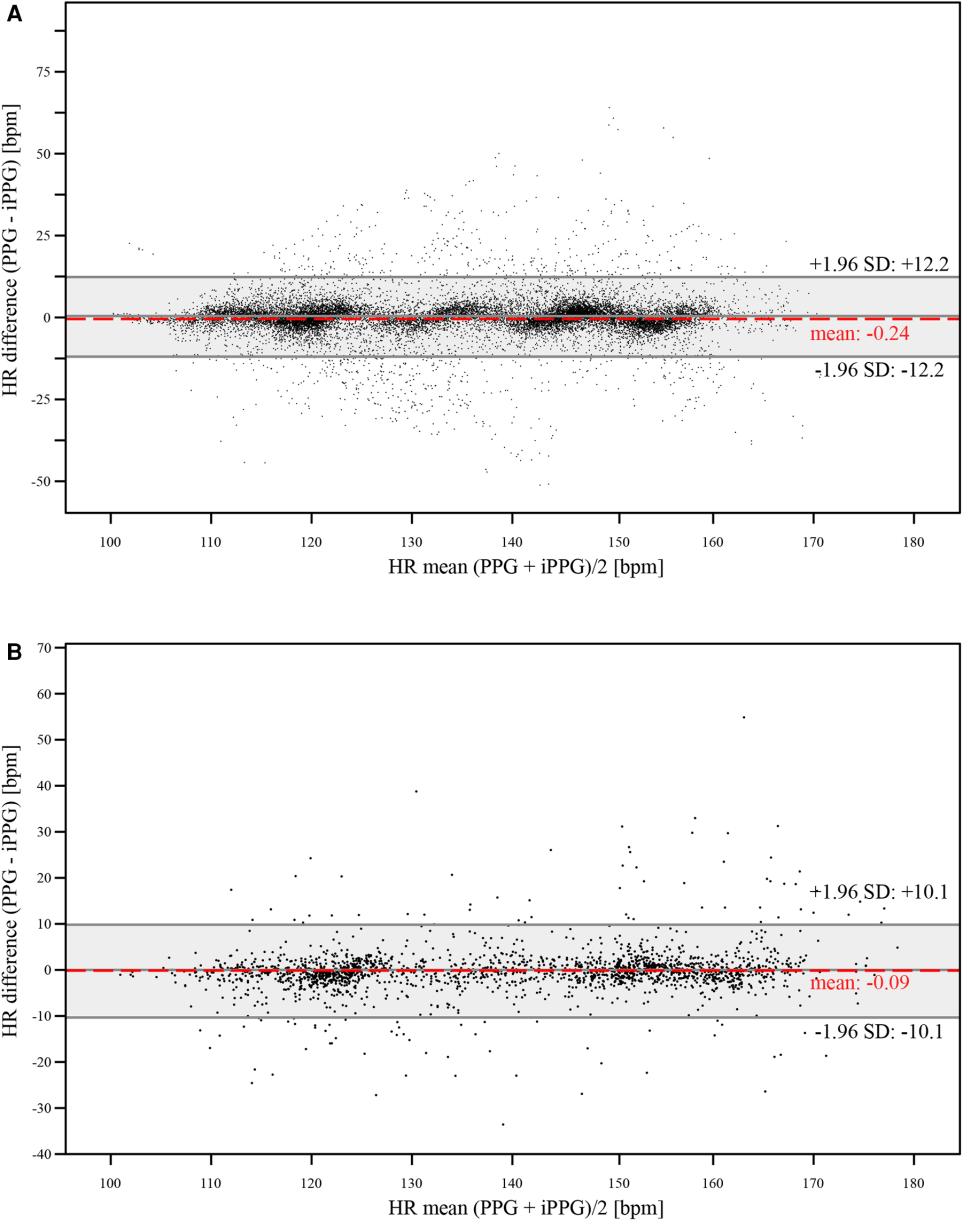
The Bland-Altman plots show the measured HR values derived from the reference and the iPPG system (**A**) with a 1 s sampling interval, (**B**) with an averaging time of 8 s. The *y*-axis shows the HR differences between the two methods in beats per minute (bpm), while the *x*-axis shows their mean HR in beats per minute (bpm).

Additionally, we assessed the level of agreement between the HR measurements obtained from the Masimo sensor and the camera-based sensor using Bland-Altman plots. Figure 3A presents the results for the 1 s sampling intervals, while Figure 3B displays those for the averaging time of 8 s.

The percentage distribution of the reasons for signal failures was summarized using relative frequencies.

The significance level was set at *α *= 0.05 for all analyses. All statistical procedures were executed using SPSS software (IBM Corp. Released 2022. IBM SPSS Statistics for Windows, Version 29.0.0.0 Armonk, NY: IBM Corp.).

## Results

At the end of the measurement phase, we collected 23 measurements of different lengths from 20 patients for evaluation. The recordings were frequently monitored during the measurement phase. For six of them, it was subsequently determined that, despite diligent preparation of the measurements, conditions for a successful acquisition of HR data were not met.

In three measurements, the ROI could not be placed accurately. The videos of two other measurements were overexposed due to strong ambient light, and the video of a third measurement was underexposed. The three cases in which the illumination conditions prevented any evaluation were carried out early in the measurement phase. The illumination conditions and video exposure were more closely monitored and regulated during subsequent measurements. The following measurements resulted in 17 recordings from 14 patients available for evaluation.

**Figure 4:**
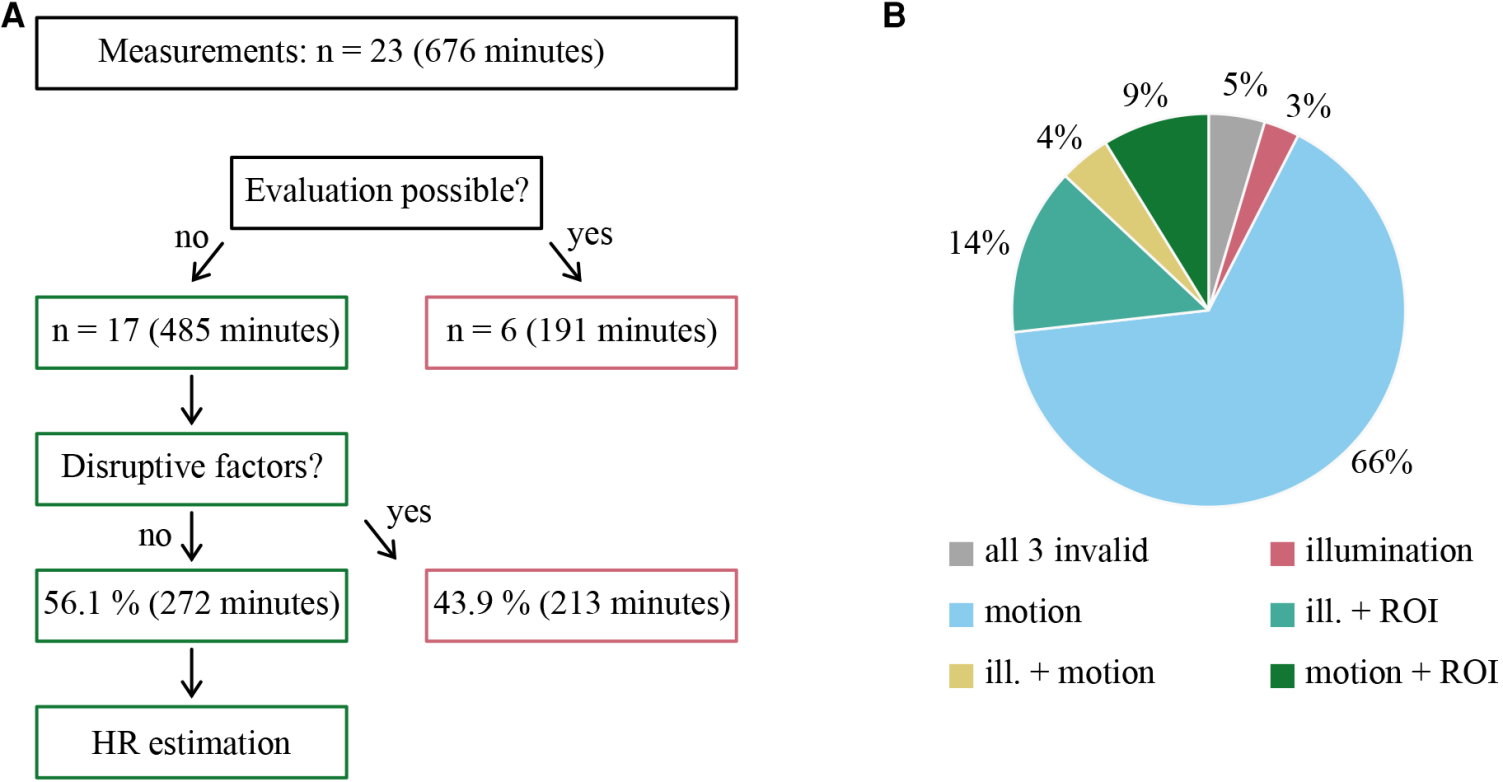
(**A**) Flowchart summarizing the selected recordings that could be evaluated, where *n* corresponds to the number of the respective measurements. (**B**) Distribution of *a priori* identified disruptive factor described in the text. These can be divided into invalidity due to motion, illumination, ROI inaccuracy, and combinations thereof.

These 17 measurements covered 485 min of data material and, thus, 71.7% of the original data volume. As mentioned, these measurements were checked for possible disruptive factors. In this analysis, 272 min of the 485 min of data material could be identified as fault-free, which corresponds to 56.1% of the evaluable data. From all originally recorded measurements, 40.2% could be evaluated. 43.9% of the evaluable data were excluded from further statistical analysis due to the presence of essentially three confounding factors. These were motion artifacts, illumination fluctuations (under- and overexposure), and loss of region of interest. As summarized by Figure 4, more than one confounding factor could be present at the same time.

Of the 14 patients whose measurements contributed to the HR evaluation, 11 were female (78.6%), while 3 were male (21.4%). All study participants exhibited characteristics consistent with individuals of Caucasian ancestry, characterized by predominantly fair or white skin pigmentation. Their corrected gestational age ranged from 27.4 to 41.9 weeks, with a mean of 33.7 weeks. Their weight at the time of measurement ranged from 1,580 to 3,710 g, with a mean weight of 2,353 g. The median HR, as measured by Masimo, was 144 beats per minute (bpm), ranging from 61 to 199 bpm. The 25th and 75th percentiles were at 125 bpm and 154 bpm, respectively. Further details are available in Table 1.

**Table 1:** The demographic and clinical characteristics of the studied population are summarized as follows.

Parameter	*N*	Mean ± SD^a^/ Median (25th–75th percentile)^b^
Gestational age [weeks]	14	33.7 ± 4.1^a^
Age [hours]	14	641.6 ± 805.4^a^
Weight [g]	14	2,352.9 ± 694.6^a^
Temperature [°C]	14	37.0 ± 0.2^a^
Bilirubin [µmol/L]	14	50.1 ± 62.5^a^
Heart rate [bpm]	14	144 (125–154)^b^

Data marked with ^a^correspond to mean ± SD. Data marked with ^b^correspond to the median (25th/75th percentile).

We compared HR data obtained from our camera-based sensor and pulse oximetry reference. We evaluated the data over 1 s sampling intervals and 8 s time averaging. The mean difference between the two methods for 1 s sampling intervals was −0.2 bpm (95% CI −0.8 to 0.4, LOA ± 12.2). For the 8 s time averaging, the mean difference was −0.09 bpm (95% CI −0.7 to 0.6, LOA ± 10.1). Notably, the differences between the methods for both sampling intervals did not achieve statistical significance (*p* = 0.396 for 1 s averaging time, respectively *p* = 0.772 for 8 s averaging time) (see Table 2).

**Table 2:** Mean HR difference in beats per minute and limits of agreement (LOA) comparing Masimo vs. iPPG.

Parameter	Estimate	95% confidence interval	*p* value	LOA
Mean difference Masimo vs. iPPG 1 s sampling intervals [bpm]	−0.2	−0.8 to 0.4	0.396	±12.2
Mean difference Masimo vs. iPPG 8 s time averaging [bpm]	−0.09	−0.7 to 0.6	0.772	±10.1

## Discussion

Our study addressed various questions assembled on the results of our pilot study (19), where the measurement accuracy of our camera-based sensor in a patient collective of healthy newborns and late premature infants was tested using two different measurement methods. The 2D measurement yielded similar results (*p* = 0.359) to the reference system, with an average difference of +3.0 bpm and a range of ±36.6 bpm. With the 3D measurement, we found that the results differed significantly from each other (*p* = 0.010), and there was a mean difference of +8.6 bpm (LOA ± 44.7 bpm). We then abandoned the 3D measurement method for further study phases.

In this study, we aimed to test the measurement accuracy of our adapted 2D system and, simultaneously, test the feasibility for longer observational periods as might be required in IMC. Therefore, we chose measurement times of 30 min and advanced to a more challenging group of patients comprised by sick newborns and premature infants who were in the neonatal IMC for therapeutic purposes.

It is important to note that we achieved a significant increase in agreement with the pulse oximetry reference, with an average difference of −0.24 bpm, with associated LOA of ±12.16 bpm. Compared to our pilot study, this is an order of magnitude more accurate.

In standard practice at the neonatal IMC, default sampling intervals are set to 8 s in order to avoid unnecessary alarms (7). For this reason, we also time averaged over 8 s and again evaluated the mean difference in the HR data between the two systems used. Using the 8 s time average further reduced the mean difference between the iPPG results and the reference to −0.09 bpm and an associated LOA of ±10.12 bpm. In this way, values that have changed significantly in the short term can better be eliminated from the analysis, and unnecessary alarm signals can be prevented.

Another question we could not answer in our pilot study was which disruptive factors influence our measurements and to what extent. In the current study, a total of 43.9% of the data that could be evaluated was perturbed by external factors. An evident and significant confounding factor is patient motion. This often includes the patient turning or covering the front of their head, leading to an invalid ROI. This accounted for most of the signal dropouts. While proper camera positioning is essential, contactless methods are susceptible to such disturbances, which can currently only be addressed procedurally and depending on the setting.

A further factor was the illumination on the station. As mentioned earlier, we did not carry out the measurements under standardized conditions. In the course of the measurement phase, we adapted the illumination as each individual situation demanded. However, under- and overexposed images still accounted for 25.5% of the data that could not be evaluated. This can be improved using adaptive exposure settings and defining limits for device operation.

While further research is needed to understand and limit the confounding factors of contactless measurement schemes, new technological opportunities arise. The camera-based approach allows the gathering of data on measurement conditions during ongoing recordings. This means that data acquired in this way can be labeled to give feedback on the validity of measurements during ongoing clinical monitoring.

Another aspect that needs to be addressed in the future is the ability of conventional contact-based monitors to measure several vital signs in parallel. In addition to HR, this also includes SpO_2_, respiratory rate, and blood pressure. However, the same confounding factors as for HR estimation will also apply to these parameters.

Finally, up to this point, our results can only be applied to newborns and premature infants who are cardiopulmonary stable. In the future, our approach should be tested in the patient group of cardiopulmonary unstable patients.

## Limitations

Our findings are subject to limitations arising from various factors, which we shall discuss below.

Firstly, it is crucial to acknowledge that the high precision of the iPPG sensor in artifact-free intervals is significantly constrained by a notably high rate of missing intervals. This limitation arose from various disruptive factors encountered during data collection, including motion artifacts, varying ambient light intensity, and loss of ROI. Additionally, user-induced errors, particularly during the initial measurement phases, contributed to this limitation.

Similar to our pilot study, we could not utilize ECG for reference due to the unavailability of devices capable of storing data at the required intervals. ECG is considered the gold standard for HR determination. A potential solution for addressing this challenge in future studies involves simultaneously recording an ECG monitor using an additional webcam.

Another limitation is that the population of this study exclusively encompassed late preterm infants and newborns that are clinically stable and do not need intensive care. Additionally, this excluded extending our dataset to extremely preterm infants or those with an unstable circulatory status. These populations need to be explored in a subsequent study within the neonatal intensive care unit.

Finally, all study participants exhibited characteristics consistent with individuals of Caucasian ancestry, characterized by predominantly fair or white skin pigmentation. Consequently, we could not assess possible inaccuracies stemming from variations in skin pigmentation.

## Conclusions

In neonatology, the accurate monitoring of vital parameters is essential for critically ill newborns. Building on a previous pilot study, we evaluated contactless imaging photoplethysmography (iPPG) within a neonatal intermediate care ward. Evaluating 20 cardiopulmonary stable, late preterm newborns and full-term compromised newborns, we compared a camera-based measurement system to a conventional pulse oximeter, considering potential artifacts like motion, variation in illumination, and accuracy of regions of interest. Although 43.9% of the data were excluded due to *a priori* evaluated confounding factors, our results demonstrated high accuracy, with mean differences of −0.2 bpm (1 s sampling) and −0.09 bpm (8 s averaging), aligning with medical standards (32). These findings support the efficacy of non-contact HR measurement in clinical settings, emphasizing the importance of *a priori* identifying potential artifacts for safer clinical measurements. While further research is warranted to expand parameter range and patient diversity, our study underscores the promising accuracy of contactless measurement schemes for neonatal vital sign monitoring.

## Data Availability

The raw data supporting the conclusions of this article will be made available by the authors, without undue reservation.
